# The effects of a globin blocker on the resolution of 3’mRNA sequencing data in porcine blood

**DOI:** 10.1186/s12864-019-6122-2

**Published:** 2019-10-15

**Authors:** Kyu-Sang Lim, Qian Dong, Pamela Moll, Jana Vitkovska, Gregor Wiktorin, Stephanie Bannister, Dalia Daujotyte, Christopher K. Tuggle, Joan K. Lunney, Graham S. Plastow, Jack C. M. Dekkers

**Affiliations:** 1 Department of Animal Science, Iowa State University, Ames, 50011 Iowa USA; 2 Lexogen GmbH, Campus Vienna Biocenter 5, 1030 Vienna, Austria; 3 USDA, ARS, BARC, Beltsville, Maryland 20705 USA; 4 University of Alberta, Edmonton, T6G 2P5 Alberta Canada

**Keywords:** Pig, Blood, 3’mRNA sequencing, Globin blocking, Gene expression

## Abstract

**Background:**

Gene expression profiling in blood is a potential source of biomarkers to evaluate or predict phenotypic differences between pigs but is expensive and inefficient because of the high abundance of globin mRNA in porcine blood. These limitations can be overcome by the use of QuantSeq 3’mRNA sequencing (QuantSeq) combined with a method to deplete or block the processing of globin mRNA prior to or during library construction. Here, we validated the effectiveness of QuantSeq using a novel specific globin blocker (GB) that is included in the library preparation step of QuantSeq.

**Results:**

In data set 1, four concentrations of the GB were applied to RNA samples from two pigs. The GB significantly reduced the proportion of globin reads compared to non-GB (NGB) samples (*P* = 0.005) and increased the number of detectable non-globin genes. The highest evaluated concentration (C1) of the GB resulted in the largest reduction of globin reads compared to the NGB (from 56.4 to 10.1%). The second highest concentration C2, which showed very similar globin depletion rates (12%) as C1 but a better correlation of the expression of non-globin genes between NGB and GB (*r* = 0.98), allowed the expression of an additional 1295 non-globin genes to be detected, although 40 genes that were detected in the NGB sample (at a low level) were not present in the GB library. Concentration C2 was applied in the rest of the study. In data set 2, the distribution of the percentage of globin reads for NGB (*n* = 184) and GB (*n* = 189) samples clearly showed the effects of the GB on reducing globin reads, in particular for *HBB*, similar to results from data set 1. Data set 3 (*n* = 84) revealed that the proportion of globin reads that remained in GB samples was significantly and positively correlated with the reticulocyte count in the original blood sample (*P* < 0.001).

**Conclusions:**

The effect of the GB on reducing the proportion of globin reads in porcine blood QuantSeq was demonstrated in three data sets. In addition to increasing the efficiency of sequencing non-globin mRNA, the GB for QuantSeq has an advantage that it does not require an additional step prior to or during library creation. Therefore, the GB is a useful tool in the quantification of whole gene expression profiles in porcine blood.

## Background

The blood transcriptome has attracted much attention in animal health and disease, as well as for humans [1], because the peripheral blood is a very informative tissue type as a source of biomarkers for prediction of pathological changes in host animals and is easily collected without having to sacrifice the animal [2]. In swine, transcriptional bloodomics based on RNA-sequencing (RNA-seq) has been applied to various disease challenge models such as porcine reproductive and respiratory syndrome virus (PRRSV) [3, 4], porcine circo virus type 2 (PCV2) [5], African swine fever virus [6], and salmonella [7].

It is widely accepted that its relatively high cost per sample is a limit to the application of standard RNA-seq to large-scale population studies of gene expression profiling. More recently, QuantSeq 3′ mRNA sequencing (QuantSeq, Lexogen, Austria) was developed as a more cost-effective approach to quantify gene expression levels in RNA samples [8]. In the QuantSeq approach, only one read per transcript, targeting the 3′ end, is generated, so that gene expression can be quantified with a much smaller number of sequencing reads per sample compared to standard RNA-seq. As a result, QuantSeq allows a high level of multiplexing of samples and greatly reduces the required sequencing depth and data processing time per sample [8]. Moreover, with QuantSeq, read counts directly reflect the level of expression of a gene, without requiring adjustment based on transcript length [9]. However, the application of QuantSeq is limited to quantifying gene expression, annotating the 3’end of transcripts, and detecting alternative polyadenylation, because QuantSeq only provides reads for the 3’end of genes.

Similar to human blood, globin mRNAs, such as *HBA* and *HBB*, account for a large proportion of mRNA in porcine blood [10, 11], leading to a decrease in the resolution of RNA-seq data from blood. To overcome this, methods to deplete globin mRNA prior to sequencing or to block the sequencing of globin mRNA have been developed. For standard RNA-seq in porcine blood, Choi et al. [10] reported that commercial globin depletion methods developed for human blood were not effective and proposed a modified RNase H mediated globin depletion protocol using porcine globin-specific oligonucleotides. Although this method reduces the abundance of globin RNA reads in porcine blood effectively, it adds steps the sample processing protocol, which compromises not only RNA quality but also increases labor, time, and costs. In addition, some non-globin genes had lower relative read counts in globin depleted samples, similar to previous reports about other globin depletion methods for human blood [12, 13]. More recently, Krjutškov et al. [14] introduced a globin mRNA locking assay for human blood, which blocks globin cDNA synthesis by adding just 10 min of incubation with globin-specific oligonucleotides, prior to sequencing. They also showed that this approach can be extended to the blood of other species such as mouse and rat.

No work has been reported on globin depletion methods for QuantSeq. Recently, novel Globin Blocker (GB) methods for QuantSeq of mRNA from human and porcine blood were commercially released (Lexogen, Austria). The porcine GB kit contains a porcine globin-specific oligonucleotide mix to block the second strand synthesis of *HBA* and *HBB* in the QuantSeq library preparation step, which makes it seamlessly compatible with the QuantSeq workflow without requiring additional reaction steps. The objectives of this study were to validate the effectiveness of QuantSeq with GB in porcine blood in terms of its specificity for *HBA* and *HBB* depletion and its impact on the discovery rate of non-globin genes, and to investigate the factors that affect the variability of its effectiveness between samples.

## Results

**Fig. 1:**
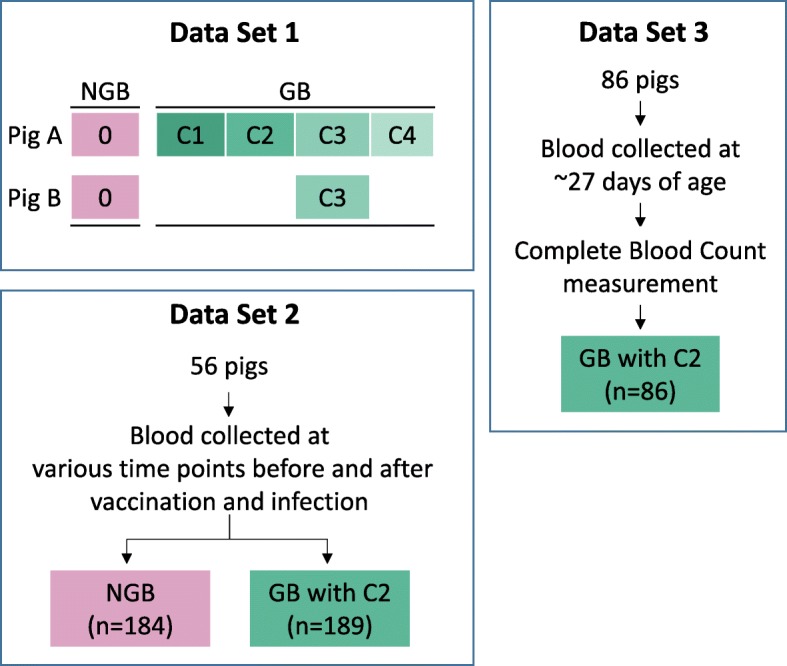
Illustration of the QuantSeq blood gene expression data sets used for analysis: (a) Data Set 1 with samples from 2 pigs without (NGB) and with (GB) inclusion of the globin blocker at 4 concentrations (C1-C4); Data Set 2 with samples from 56 pigs at multiple time points before and after vaccination and infection with porcine reproductive and respiratory syndrome virus; and Data Set 3 with samples collected on 86 young healthy pigs

In order to evaluate the effectiveness and the effects of the GB, QuantSeq data from three independent data sets were used, as illustrated in Fig. 1: 1) technical replicates of a blood sample from each of two pigs to evaluate different concentrations of the GB, 2) biological replicates consisting of 373 blood samples from 56 pigs to evaluate variation in globin depletion, and 3) biological replicates consisting of blood samples from 86 pigs to identify factors that affect the efficiency of globin depletion by the GB. The first data set was generated specifically for this study, while the second and third data sets were generated for general gene expression studies.

### Effect of GB and its concentration on QuantSeq globin reads (data set 1)

To investigate whether the GB is effective in reducing globin reads in QuantSeq results, a total of 7 aliquoted RNA samples from two weaned pigs were used in QuantSeq library construction. Each library was sequenced on two lanes. On average, 31.82 ± 3.48 million (M) clean reads after trimming the raw QuantSeq reads were obtained per RNA sample, ranging from 27.05 to 36.23 M (Table 1). Mapping rates to the pig reference genome 11.1 were similar between samples (~ 98%). The GB libraries showed lower proportions of unique-mapping reads (up to 67.7%) than the non-GB (NGB) libraries (up to 78.3%) and, consequently, the proportions of reads mapped to gene regions were also lower for the GB libraries.

**Table 1:** The effects of inclusion of a globin blocker in library construction on QuantSeq 3′ mRNA sequencing data in data set 1

Biological replicate	Globin blocker concentration	Read counts by genes (millions)							Number of expressed genes ^b^
		Total reads ^a^	Aligned reads	Uniquely mapped reads	Gene reads	HBA reads	HBB reads	Non-globin reads	
A	0	33.56	33.03 (98.41)	26.29 (78.32)	22.91 (68.25)	6.50 (19.38)	12.43 (37.02)	3.98 (11.85)	8437
	C1	36.23	35.44 (97.81)	22.31 (61.57)	13.96 (38.54)	2.76 (7.61)	0.92 (2.55)	10.28 (28.38)	9612
	C2	31.65	31.08 (98.20)	19.17 (60.57)	12.10 (38.21)	3.13 (9.87)	0.67 (2.10)	8.30 (26.24)	9692
	C3	27.05	26.48 (97.91)	16.83 (62.21)	11.44 (42.30)	4.26 (15.73)	0.96 (3.54)	6.23 (23.02)	9516
	C4	32.92	32.32 (98.17)	22.28 (67.66)	16.49 (50.08)	5.29 (16.07)	4.50 (13.67)	6.70 (20.34)	9270
B	0	34.12	33.44 (98.00)	25.19 (73.82)	23.68 (69.39)	8.77 (25.70)	11.54 (33.82)	3.37 (9.87)	7090
	C3	27.23	26.67 (97.92)	13.33 (48.96)	11.00 (40.38)	4.87 (17.88)	0.91 (3.34)	5.22 (19.15)	8547

Abbreviations: *HBA* hemoglobin subunit alpha, *HBB* hemoglobin subunit beta

The numbers in parentheses refer to percentages of the total reads (%)

^a^The number of reads after trimming by Bbduk

^b^Threshold of expressed gene: ≥ 5 reads counts per 10 millions of total clean read counts

The effects of GB on the proportion of globin reads were investigated using a general linear model with the fixed effects of biological replicate and GB treatment based on the experimental design (Table 1). The proportions of globin gene reads were significantly lower in the GB compared to the NGB samples (Additional file 1: Table S1); globin reads occupied 58.0% of the total clean read counts in the NGB samples but only 19.5% in the GB samples (*P* = 0.005). In particular, *HBB* reads, which were predominant in the NGB samples (35.4%), were dramatically reduced in the GB samples (to 4.3%, *P* = 0.002). The *HBA* read proportion also tended to be lower in the GB samples (*P* = 0.085). As a result, the proportion of reads mapped to non-globin gene regions, which are the reads of interest, was about twice as large for the GB samples (22.2%) compared to the NGB samples (10.9%, *P* = 0.016).

**Fig. 2:**
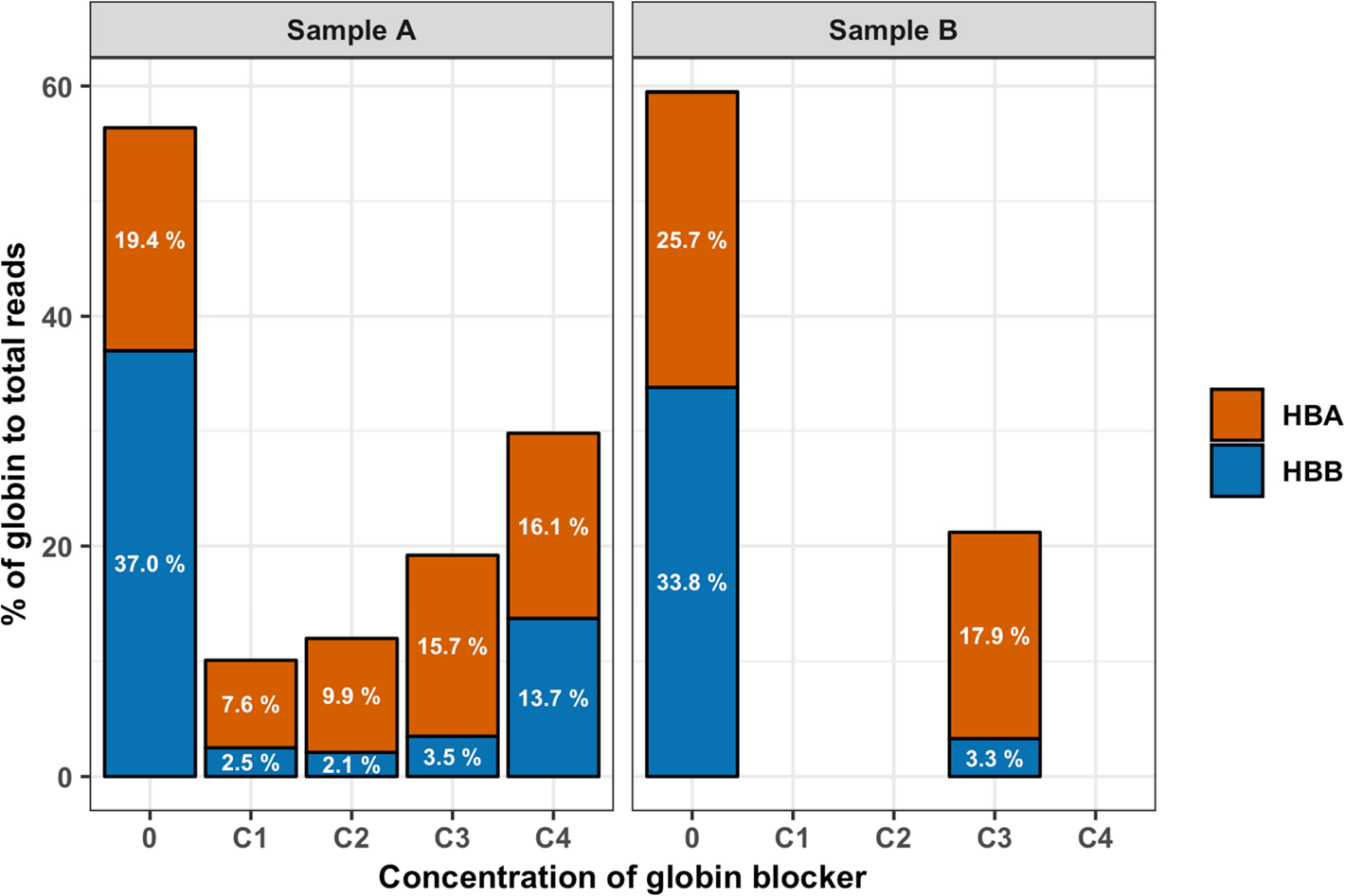
The effects of the globin blocker on the percentage of globin reads

**Fig. 3:**
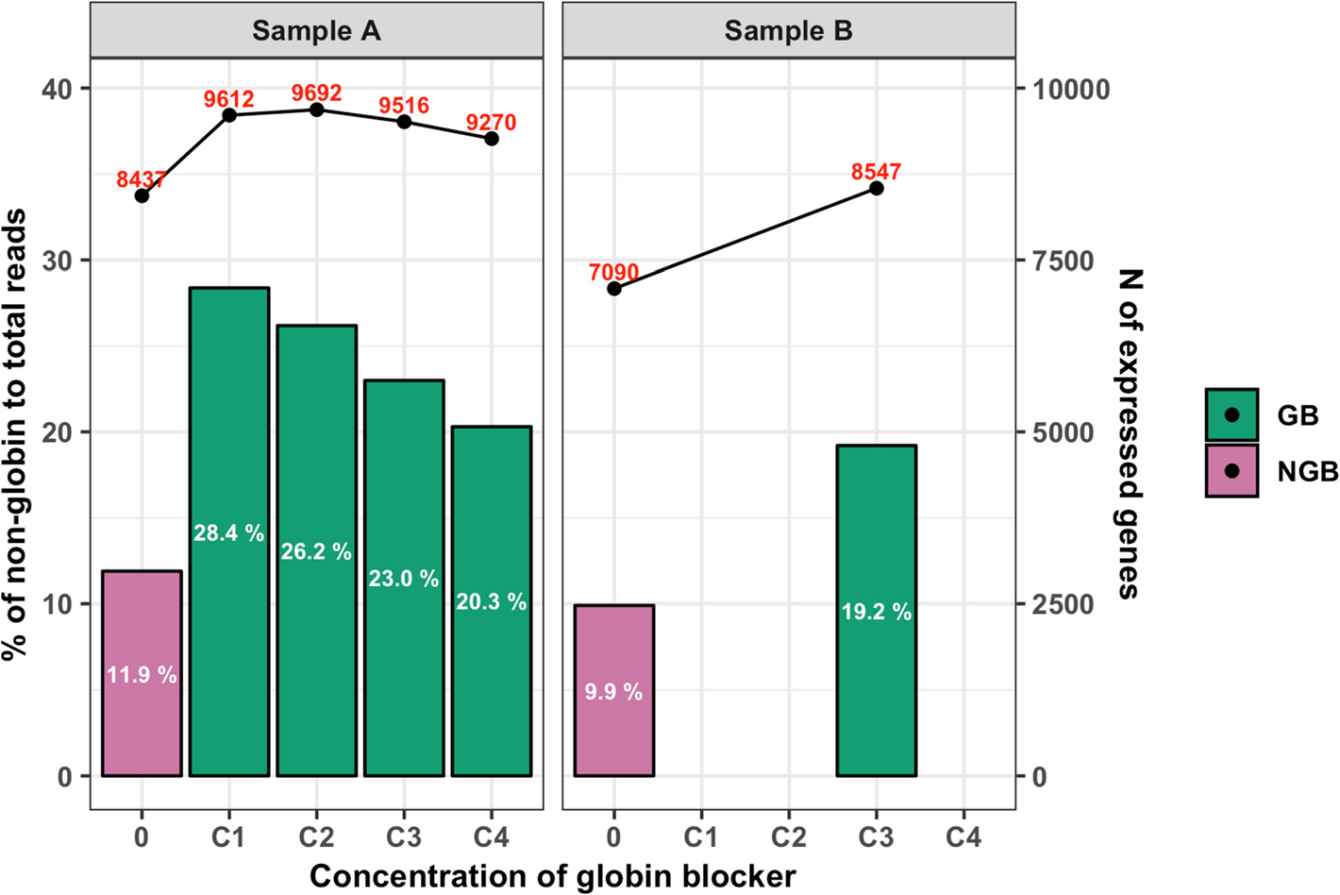
Percentage of non-globin gene reads to the number of total clean reads and the number of reliably detected genes

Although the GB showed a clear effect on globin depletion, its effectiveness differed between the four serially decreased GB concentrations (C1 to C4) that were used, as shown in Table 1 and Fig. 2. Depletion of *HBA* reads was most effective for C1 and C2, while the number of *HBB* reads was reduced very effectively with all concentrations except C4. Overall, C1 and C2 showed the highest efficiency in globin depletion (10.1 and 12.0%), resulting in the highest proportions of non-globin reads (28.4 and 26.2%; Table 1 and Fig. 3).

### Effects of GB on gene expression profiles (data set 1)

**Fig. 4:**
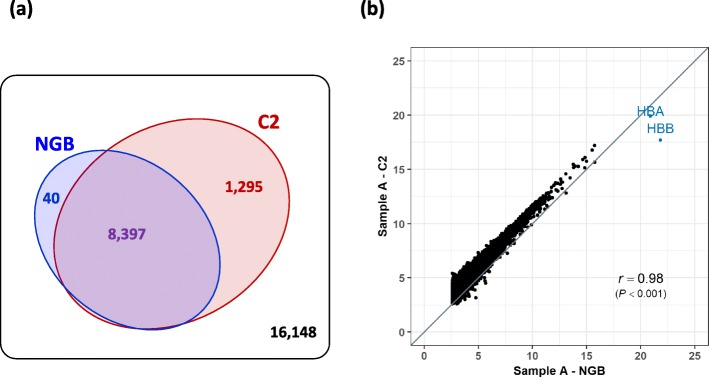
The effects of the globin blocker at concentration C2 on the levels of gene expressions* in sample A. **a** Venn diagram of number of detected expressed genes in the NGB and GB (**b**) Scatter plot of counts per 10 million reads between the NGB and GB. *log2(count per 10 million + 1)

To compare the gene expression levels between NGB and GB samples, raw gene counts were scaled to counts per 10 M of total clean reads for each sample, rather than the typically used count per M of reads mapped to the genes, because the number of reads mapped to the genes varied between GB and NGB samples and also between GB concentrations. The number of reliably expressed genes, which was based on having a scaled count of 5 or greater, following Choi et al. [10], differed between biological replicates but was greater for the GB samples than for the NGB samples (Table 1 and Fig. 3). Figure 4 shows details of the gene expression profiles for the NGB and GB sample with concentration C2, which had the highest number of detected genes in biological replicate A. Among 25,880 total genes, 8397 genes were expressed in both the NGB and GB sample. Scaled counts of non-globin genes for the NGB and GB sample were highly correlated (*r* = 0.98; *P* < 0.001), which was greater than that of C1 (*r* = 0.96, *P* < 0.001). When plotting scaled counts for the GB sample against those of the NGB sample (Fig. 4), counts for most genes fell above the diagonal, which indicates that the scaled counts of these genes was greater in the GB sample. *HBA* and *HBB* still had the highest scaled counts in the GB sample but they were a factor 2.0 and 18.3 lower, respectively, than in the NGB sample. In the GB sample, 1295 genes were detected that were not detected in the NGB sample, while only 40 genes were detected in only the NGB samples (Fig. 4 and Additional file 1: Table S3). Similar trends in the number of detectable genes and the correlation of scaled counts between NGB and GB samples were also observed for the other GB concentrations (Additional file 1: Table S2 and Additional file 2: Figure S1). Sequence similarity of non-globin genes that were detected only in NGB samples with *HBA* and *HBB* was investigated to identify possible non-specific hybridization, but no evidence for this was found. These results demonstrate that GB enhanced the coverage of non-globin genes in the RNA sequence reads and increased the overall number of read counts that were assigned to non-globin genes with QuantSeq. Taken together, GB with concentration C2 as shown in Additional file 2: Figure S2 had similar globin depletion efficiency as C1 but showed better gene coverage and a greater consistency of observed expression levels of non-globin genes with those from NGB, and was used to generate data sets 2 and 3.

### Variation in remaining globin reads in GB samples (data set 2)

**Fig. 5:**
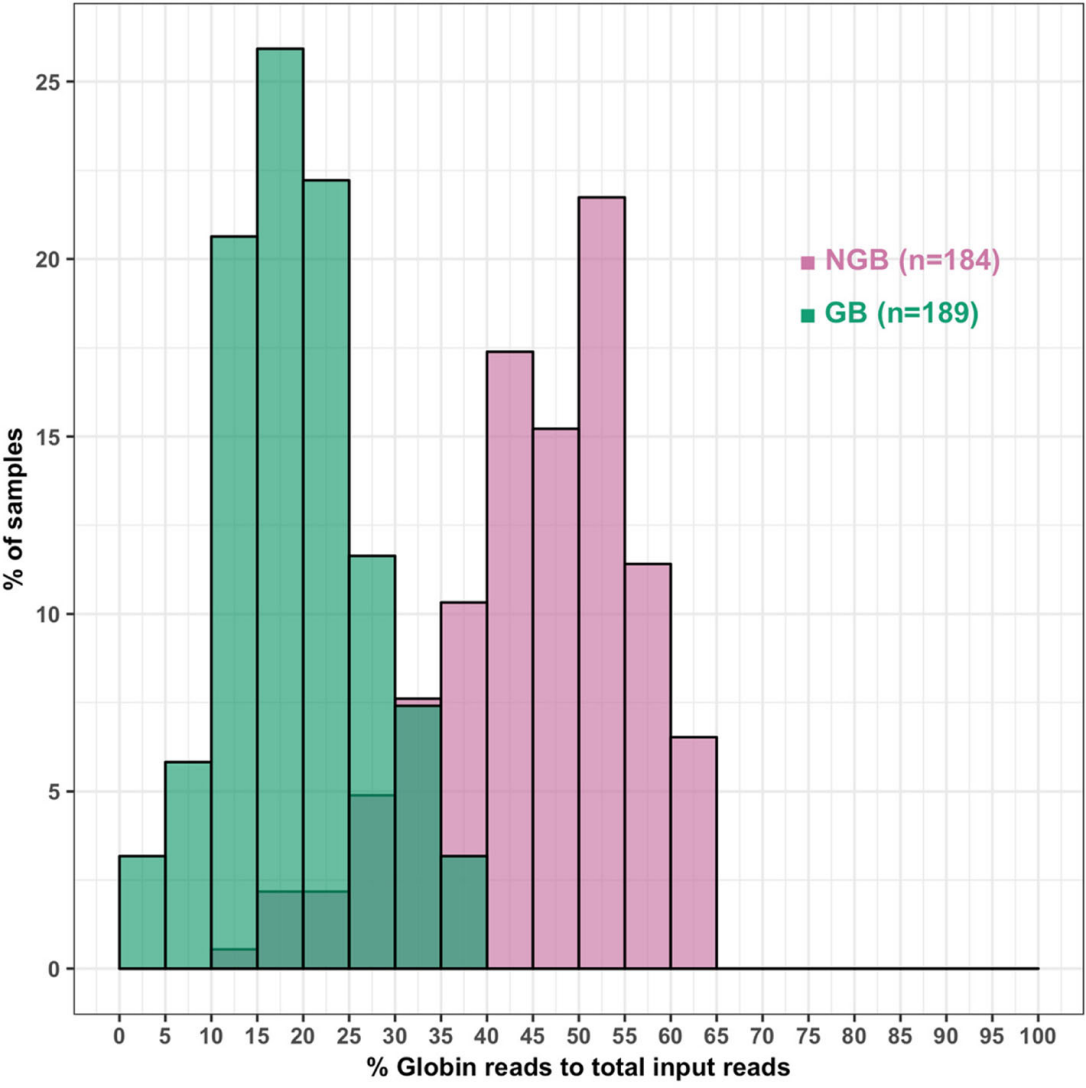
Distributions of globin read counts as a percentage of total reads in biological replicates by NGB and GB

Next, we evaluated the effect of GB on globin depletion using large population-level QuantSeq data from the biological replicates in data set 2. Blood RNA samples from the PRRS Host Genetics Consortium (PHGC) trials described in [15] were used in the construction of the QuantSeq libraries for NGB (*n* = 184) and GB (*n* = 189). The constructed libraries were multiplexed for up to 96 samples and sequenced on one lane. On average, 3.38 M and 3.28 M of total clean reads were generated for the NGB and GB samples, respectively. Consistent with data set 1, the NGB and GB samples had similar alignment rates (%) but the NGB samples had a higher unique-mapping rate and gene read % than the GB samples (Additional file 1: Table S4). Distributions of globin read proportions clearly showed that the GB substantially reduced globin reads (Fig. 5). In particular, the proportion of *HBB* was much lower in the GB samples (Additional file 1: Table S4 and Additional file 2: Figure S3), similar to what was observed for data set 1. Similar to the NGB samples, the GB samples also showed large variation in the proportion of globin reads present. Note that the NGB and GB samples in this data set came from two different gene expression profiling studies. Nevertheless, the mRNA levels of *HBA* and *HBB* in the original GB samples likely also varied widely, similar to what was observed for the NGB samples, and this may be the most important factor affecting the proportions of *HBA* and *HBB* reads still present in the GB QuantSeq reads. We did, however, use these data to investigate other factors that may have affected the number of globin reads that remained in the GB samples, including RNA quality, as quantified by RNA integrity number (RIN), and sequencing depth based on the number of total reads (Additional file 2: Figure S4). The percentage of globin reads in the GB samples showed a significant correlation with RIN (*r* = 0.30 and *P* < 0.001) and sequencing depth (*r* = − 0.20 and *P* = 0.007). These same relationships were less strong in the NGB samples (RIN, *r* = 0.13 and *P* = 0.080; sequencing depth, *r* = − 0.02 and *P* = 0.800). Although the percent of globin reads in the GB samples appeared to be affected by RIN and sequencing depth, these factors only account for 13% of the variation in the percentage of globin reads in the GB samples (*R*^2^ = 0.13). The proportion of globin mRNA in the original sample probably explained a large portion of the remaining variation but, as indicated, this could not be investigated in this data set.

### The effect of hemoglobin concentration and reticulocyte count in blood on effectiveness of GB (data set 3)

To address the effect of hemoglobin concentration (g/L) and reticulocyte count (10^3^/μL) in the original sample on the proportion of globin reads that remained in GB samples, we used data from samples for which measurements of complete blood counts were available. RNA samples (*n* = 86) were extracted from blood samples collected at ~ 27 days of age. The hemoglobin concentration and reticulocyte count were measured by a flow cytometry-based hematology analyzer [16]. QuantSeq libraries were constructed with GB concentration C2 and multiplexed for sequencing on one lane. On average, 7.29 M clean reads were obtained per sample and of which 10.7% were globin (Additional file 1: Table S5). We used a general linear model for analysis, with RIN, sequencing depth, hemoglobin concentration, and reticulocyte count as covariates. Only reticulocyte count showed significant (*P* < 0.001) and positive relationships with the percentage of globin reads, as well as with the percentages of *HBA* and *HBB* reads (Table 2). Reticulocyte count explained 26.6% of the variance in the percentage of globin reads, in addition to 10.6% of the variation explained by RIN and library size in this data set. Hemoglobin concentration did not have significant associations with the percentage of globin reads in the GB samples.

**Table 2:** The linear regression results for the globin read percent in data set 3

	Regression coefficient and SE of covariates				
Dependent variable	RIN	Library size (M)	Hemoglobin concentration (g/L)	Reticulocyte count (10^3^/μL)	Adjusted *R*^2^
Globin reads (%)	−0.43 (0.35)	−0.26 (0.34)	− 0.03 (0.04)	0.04 (0.01) ^***^	0.34 ^***^
Hemoglobin alpha reads (%)	−0.40 (0.30)	0.01 (0.29)	−0.03 (0.03)	0.03 (0.01) ^***^	0.27 ^***^
Hemoglobin beta reads (%)	−0.03 (0.14)	−0.27 (0.14)	0.01 (0.02)	0.01 (< 0.01) ^***^	0.23 ^***^

The level of significance: * *P* < 0.05; ** *P* < 0.01; *** *P* < 0.001

The numbers in parentheses refer to standard errors

## Discussion

Blood has long been used as a diagnostic source for both humans and animals because it reflects the status of the subject at the time of sampling. For the same reason, blood gene expression profiling is a critical tool for understanding host genetics of diseases, as well as a source of biomarkers to predict susceptibility or resilience to disease. However, porcine blood samples have a unique limitation that is caused by the amount of globin mRNA that can be present, which is an issue for both RNA sequencing, as well as for microarray-based gene expression profiling. The amount of globin mRNA in blood varies between species. Correia et al. [11] showed that bovine and equine blood contain very low levels of globin mRNA transcripts compared to human and porcine blood, which was consistent with a previous report on porcine blood (~ 46%) [10]. We also observed similar proportions of globin reads in QuantSeq data from the NGB samples in both data set 1 (*n* = 2; 56.4 and 59.5%) and data set 2 (*n* = 184; average 47.8 ± 11.3%).

In data set 1, we first tested whether GB had an effect on the globin read proportion compared to NGB samples using a linear model. Then, a comparison of gene expression levels depending on the GB concentrations was conducted based on the proportion of non-globin to total reads and the number of reliably detected genes. The GB applied in QuantSeq library construction successfully reduced the proportions of *HBA* and *HBB* reads and showed optimum efficiency with concentrations C1 and C2 of the globin blocker compared to higher concentrations (Fig. 2). The GB more than doubled the proportion of reads that mapped to non-globin genes and this led to an increase in the number of detected genes (Fig. 3), which agrees with previous studies on globin depletion in RNA-seq [10, 12–14]. There were, however, some genes which were detected in the NGB samples but not in the corresponding GB samples, but these genes did not show sequence similarity with *HBA* or *HBB*. Similar results were reported for previous RNA-seq globin depletion methods [10, 12, 13]. The assigned reads for these genes were, however, very low in the NGB samples and were also present qualitatively in the GB samples (Additional file 1: Table S3), although they did not meet the criteria of a reliably expressed gene in the GB samples. Therefore, the fact that they were not detected in the GB samples likely was due to sampling effects and their low count is not expected to bias the relative counts for other genes. The very high correlation of gene expression levels obtained with and without GB for the technical replicates in data set 1 further demonstrated that the GB did not introduce a significant bias in quantifying the level of expression of genes other than globin (Fig. 4). Thus, the GB for porcine blood QuantSeq meets the expected benefits that it selectively reduces *HBA* and *HBB* reads without affecting quantification of the relative expression of other genes. We also validated the lower globin read proportions in GB samples from a larger data set (Fig. 5 and Additional file 1: Table S5).

The GB samples, which had higher percentages of non-globin gene reads than NGB samples, showed lower proportions of unique-mapping reads and gene reads to total reads, as well as lower globin read percentages compared to NGB samples across all data sets (Table 1, Additional file 1: Tables S4, S5, and Additional file 2: Figure S2). This results from the sequencing space that is freed up in the GB samples after blocking globin reads being taken up by not only non-globin gene reads but also the other reads that map to multiple-regions and/or to intergenic regions. The proportion of *HBA* reads was similar in the GB and NGB samples, although the GB was designed to block both *HBA* and *HBB*. This was due to the predominant proportion of *HBB* mRNA in the original RNA samples, i.e. the GB did substantially reduce the amount of *HBA* reads relative to non-globin reads.

Previous studies have shown that globin depletion methods in RNA sequencing such as GLOBINclear™ (ThermoFisher), the modified GR protocol (Affymetrix), and GlobinLock are effective in reducing globin mRNA in blood [10, 12–14]. However, these studies used a small number of samples and could not validate that the globin depletion method worked uniformly across samples. We used data from a large-scale gene expression study generated using QuantSeq with GB (*n* = 275) to show that the percentage of globin reads still varied substantially between samples following GB, ranging from 2.3 to 37.9%. Hence, we investigated possible factors that affect the effectiveness of the GB. RNA quality and library size showed weak and moderate significant correlations, respectively, with the globin read percentage in the GB samples in data set 2, but these effects were not significant in the regression analysis that also included hemoglobin concentration and reticulocyte count in data set 3. Most importantly, the proportions of total globin, *HBA*, and *HBB* reads in the GB samples were significantly associated with reticulocyte count (*P* < 0.001) but not with hemoglobin concentration in the original sample. Hemoglobin, as well as the erythrocytes that contain it, do not have nuclei. However, reticulocytes, which are immature erythrocytes formed in the bone marrow, have nuclei, circulate in the blood, and have been shown to contribute mostly to the abundant globin mRNA levels in blood [17, 18]. These results indicate that the globin read proportions that remain in GB samples are highly correlated with the amount of globin mRNA in the original sample based on reticulocyte counts.

Overall, our results supported the benefit of GB for QuantSeq for porcine blood. However, there is another important item that should be investigated in further studies, which is that more than 16,000 genes were not detected to be reliably expressed genes in both the NGB and GB samples in data set 1 and the proportion of reads mapped to non-globin gene regions was still low (~ 35%) in the GB samples from all three data sets. Although these genes may be expressed, their reads may not be counted because of incomplete 3′ UTR annotations of the reference genome or because of multi-mapping issues for genes that have highly homologous 3′ ends.

## Conclusions

In QuantSeq from porcine blood, the GB effectively blocked globin mRNA from being sequenced, with minimal effects on the relative read counts among non-globin genes and, therefore, increased the sensitivity for detecting genes with lower expression for a given amount of sequencing. The effectiveness of the GB was validated in a large scale QuantSeq data set, which showed that differences in the proportion of globin reads that were still present in the GB samples were closely related with the initial reticulocyte count in porcine blood. The GB for QuantSeq has the advantage of seamless integration in the QuantSeq library prep workflow without impact on labor and costs because the application of GB simply requires replacing the RS solution by GB-RS solution. Taken together, this study demonstrates that QuantSeq with GB is an effective method for blood transcriptomics studies in pigs, especially for generating large-scale expression data sets.

## Methods

### 3′ mRNA sequencing data sets

Blood samples were collected in Tempus Blood RNA Tubes (Thermo Fisher Scientific, USA) and then stored at − 80 °C until RNA extraction. The RNAs were isolated using Preserved Blood RNA Purification Kit I (Norgen, Canada) according to the manufacturer’s instructions and the RIN of each extracted RNA was assessed by the 2100 Bioanalyzer (Agilent Technologies, USA) using Eukaryote total RNA 6000 Nano kit.

For data set 1, one blood sample from each of two Large-White female pigs at weaning (27 days of age) were used. The extracted RNA samples were aliquoted as technical replicates. Two aliquots from the first RNA sample A with lower RIN (3.6) were each assigned to five treatments, including NGB and GB at 4 different concentrations: C2, C3, and C4 were 1:10 dilution series and C1, which was the highest concentration, which was 5× as concentrated as C2. Because of its limited concentration, the aliquots of the second RNA sample B, which had higher RIN (4.9), was processed only with NGB and C3. The GB solutions of different concentrations were provided by Lexogen. The currently commercially available GB kit contains concentration C2.

Data set 2 consisted of available data from 184 RNA samples from the PHGC trial 16 for NGB and from 189 RNA samples from trial 20 for GB. The crossbred pigs (*n* = 56) in these two trials shared a similar genetic background and came from the same multiplier herd. The experimental design and blood sampling protocols of these two trials were described in [15]. Briefly, the pigs were vaccinated with a commercial PRRS modified live virus vaccine (Ingelvac PRRS®, Boehringer Ingelheim Vetmedica Inc., St. Joseph, MO) at around 3 weeks of age and, 4 weeks later, co-infected with field isolates of PRRS virus and porcine circovirus type 2b. Blood samples were collected at 4, 7, 11, and 14 day post vaccination and at 0, 4, 7, 11, 14, 28 days post co-infection.

Data set 3 consisted of RNAseq data on weaned barrows (*n* = 86) from two healthy multiplier farms from PigGen Canada, which were moved to a research facility in Québec, Canada, as described in [19]. The blood samples for RNA extraction were collected at ~ 27 days of age during acclimation in a quarantine nursery.

### Library construction and 3′ mRNA sequencing

RNA-seq libraries from data set 1 were generated from 100 ng, while all other libraries were generated from ~ 500 ng of total RNA using the QuantSeq 3′ mRNA-Seq Library Prep Kit FWD for Illumina (Lexogen, Austria), according to the manufacturer’s protocol. The first-strand cDNA was synthesized by reverse transcription with oligo-dT priming. The regular Removal Solution was used for NGB samples and this was replaced by RNA Removal Solution-Globin Block, *Sus scrofa* (SSC; commercially available as RS-GBSs: Lexogen Cat. No. 071) for the GB treated samples prior to the second strand synthesis, which contains porcine globin-specific oligonucleotide mixtures that bind to the first strands generated from mRNAs of *HBA* and *HBB* and, thereby, prevent second strand synthesis. To generate data set 1, a total of 14 QuantSeq libraries were multiplexed in a shared lane (34 samples in total) and sequenced with single-end 75 nucleotides using the Illumina NextSeq 500 Sequencing System (Illumina, USA). For data set 2 (*n* = 373) and data set 3 (*n* = 84), the constructed QuantSeq libraries were multiplexed using mRNA from up to 96 samples and sequenced with single-end 50 nucleotides using the Illumina HiSeq 3000 Sequencing System (Illumina, USA).

### RNA-seq analysis

The raw QuantSeq reads were trimmed using BBDuk (https://jgi.doe.gov/data-and-tools/bbtools/bb-tools-user-guide/bbduk-guide/) to remove adapter sequences, poly-A tails, and low-quality bases. Trimmed reads with a length less than 20 bp were also filtered out. Read quality before and after trimming was checked using FASTQC 0.11.5 [20]. Trimmed reads were mapped to the SSC11.1 reference genome sequence (Ensemble, http://www.ensembl.org/) using STAR 2.5.3a [21] and read counts per gene from uniquely mapped reads were calculated by HTSeq-count 0.9.1 [22] with the pig genome GTF of Ensemble release 92. The Porcine *HBA* gene (ENSSSCG00000007978) has two regions with highly similar sequences in SSC11.1 and, as a result, most *HBA* reads were classified as multiple-mapping and eliminated by HTSeq-count. To address this and count each *HBA* region only once, one of the *HBA* regions (Chr3: 41,482,260 - 41,487,800 bp in SSC11.1) was masked before alignment. The region of the *HBB*-like gene ENSSSCG00000014727, which has similar sequences to *HBB* (ENSSSCG00000014725) was also masked to avoid the multiple-mapping issue for *HBB*.

### Complete blood count measurement

Complete blood counts were available on the samples used in data set 3. Complete blood count measurement was performed using the flow cytometry-based hematology analyzer [16] according to the manufacturer’s instructions. The hemoglobin concentration (g/L) and the reticulocyte count (10^3^/μL) were used in the analysis of data set 3.

### Statistical analyses

To determine whether the globin read percent was statistically different between the NGB and GB samples of data set 1, the following general linear model was used: *y*_*ijk*_ = *GB*_*i*_ + *BioRep*_*j*_ + *e*_*ijk*_, where *y*_*ijk*_ is the observed proportion of globin reads to total reads, *GB*_*i*_ is the fixed effect of GB treatment (NGB and GB), *BioRep*_*j*_ is a fixed effect for biological replicates (pigs A and B), and *e*_*ijk*_ is a random residual. Relationships of the gene expression levels between NGB and GB at a given concentration were quantified using Pearson correlation coefficients. In data set 3, the following general linear model was used to examine the factors that contributed to variation in globin read proportions in the GB samples: *y*_*ijklm*_ = *RIN*_*i*_ + *Lib*_*j*_ + *HC*_*k*_ + *RC*_*l*_ + *e*_*ijklm*_, where *y*_*ijklm*_ is the observed proportion of globin reads to total reads, *RIN*_*i*_, *Lib*_*j*_, *HC*_*k*_, *RC*_*l*_ are covariates for RIN score, library size (M), hemoglobin concentration (g/L), and reticulocyte count (10^3^/μL), respectively, and *e*_*ijklm*_ is a random residual.

## Supplementary information

**Additional file 1: Table S1.** The effects of inclusion of a globin blocker in library construction on the composition of reads mapped to genes in data set 1. **Table S2.** The number of expressed genes in libraries created with and without globin blocker in data set 1. **Table S3.** The list of genes that were identified as expressed in the library constructed without a globin blocker but not in libraries constructed with the globin blocker at concentration C2 in sample A. **Table S4.** Summary statistics for QuantSeq 3′ mRNA sequencing data from biological replicates in data set 2. **Table S5.** Summary statistics for QuantSeq 3′ mRNA sequencing of libraries created with the globin blocker in data set 3

**Additional file 2: Figure S1.** Scatter plots of scaled counts* between NGB and GB. (a) relationship between NGB and GB with C1 in sample A, (b) relationship between NGB and GB with C3 in sample A, (c) relationship between NGB and GB with C4 in sample A, (d) relationship between NGB and GB with C3 in sample B. *log2(count per 10 million + 1). **Figure S2.** Summary statistics for QuantSeq with or without GB at concentration C2. ALIGN, aligned reads; UNIQ, unique-mapping reads; GENE, reads mapped to all genes; HBA/HBB, reads mapped to HBA and HBB genes; Non-globin, reads mapped to non-HB genes. **Figure S3.** Distributions of HBA and HBB counts as a percentage of total reads in biological replicates by NGB and GB. **Figure S4.** Scatter plots of globin read percentage (%) with RIN and the number of clean reads in NGB (a and b) and GB (c and d)

## Data Availability

Because the data were generated on samples from commercially owned animals, the data analysed in the current study are not publicly available but they can be made available by the corresponding author on reasonable request.

## Abbreviations

C: GB concentration
GB: globin blocker
M: million
NGB: non-GB
PCV2: porcine circo virus type 2
PHGC: PRRS Host Genetics Consortium
PRRSV: porcine reproductive and respiratory syndrome virus
QuantSeq: QuantSeq 3’mRNA sequencing
RIN: RNA integrity number
RNA-seq: RNA-sequencing

## References

[1] Chaussabel D (2015). Assessment of immune status using blood transcriptomics and potential implications for global health. Semin Immunol.

[2] Mohr S, Liew CC (2007). The peripheral-blood transcriptome: new insights into disease and risk assessment. Trends Mol Med.

[3] Arceo ME, Ernst CW, Lunney JK, Choi I, Raney NE, Huang T, Tuggle CK, Rowland RR, Steibel JP (2012). Characterizing differential individual response to porcine reproductive and respiratory syndrome virus infection through statistical and functional analysis of gene expression. Front Genet.

[4] Wilkinson JM, Ladinig A, Bao H, Kommadath A, Stothard P, Lunney JK, Harding JC, Plastow GS (2016). Differences in whole blood gene expression associated with infection time-course and extent of fetal mortality in a reproductive model of type 2 porcine reproductive and respiratory syndrome virus (PRRSV) infection. PLoS One.

[5] Li Y, Liu H, Wang P, Wang L, Sun Y, Liu G, Zhang P, Kang L, Jiang S, Jiang Y (2016). RNA-Seq analysis reveals genes underlying different disease responses to porcine circovirus type 2 in pigs. PLoS One.

[6] Jaing C, Rowland RRR, Allen JE, Certoma A, Thissen JB, Bingham J, Rowe B, White JR, Wynne JW, Johnson D (2017). Gene expression analysis of whole blood RNA from pigs infected with low and high pathogenic African swine fever viruses. Sci Rep.

[7] Huang TH, Uthe JJ, Bearson SM, Demirkale CY, Nettleton D, Knetter S, Christian C, Ramer-Tait AE, Wannemuehler MJ, Tuggle CK (2011). Distinct peripheral blood RNA responses to Salmonella in pigs differing in Salmonella shedding levels: intersection of IFNG, TLR and miRNA pathways. PLoS One.

[8] Moll P, Ante M, Seitz A, Reda T (2014). QuantSeq 3′ mRNA sequencing for RNA quantification. Nat Methods.

[9] Ma F, Fuqua BK, Hasin Y, Yukhtman C, Vulpe CD, Lusis AJ, Pellegrini M (2019). A comparison between whole transcript and 3′ RNA sequencing methods using Kapa and Lexogen library preparation methods. BMC Genomics.

[10] Choi I, Bao H, Kommadath A, Hosseini A, Sun X, Meng Y, Stothard P, Plastow GS, Tuggle CK, Reecy JM (2014). Increasing gene discovery and coverage using RNA-seq of globin RNA reduced porcine blood samples. BMC Genomics.

[11] Correia CN, McLoughlin KE, Nalpas NC, Magee DA, Browne JA, Rue-Albrecht K, Gordon SV, MacHugh DE (2018). RNA sequencing (RNA-Seq) reveals extremely low levels of reticulocyte-derived globin gene transcripts in peripheral blood from horses (Equus caballus) and cattle (Bos taurus). Front Genet.

[12] Mastrokolias A, den Dunnen JT, van Ommen GB, t hoen PA, van Roon-Mom WM (2012). Increased sensitivity of next generation sequencing-based expression profiling after globin reduction in human blood RNA. BMC Genomics.

[13] Shin H, Shannon CP, Fishbane N, Ruan J, Zhou M, Balshaw R, Wilson-McManus JE, Ng RT, McManus BM, Tebbutt SJ (2014). Variation in RNA-Seq transcriptome profiles of peripheral whole blood from healthy individuals with and without globin depletion. PLoS One.

[14] Krjutskov K, Koel M, Roost AM, Katayama S, Einarsdottir E, Jouhilahti EM, Soderhall C, Jaakma U, Plaas M, Vesterlund L (2016). Globin mRNA reduction for whole-blood transcriptome sequencing. Sci Rep.

[15] Dunkelberger JR, Serao NV, Niederwerder MC, Kerrigan MA, Lunney JK, Rowland RR, Dekkers JC (2017). Effect of a major quantitative trait locus for porcine reproductive and respiratory syndrome (PRRS) resistance on response to coinfection with PRRS virus and porcine circovirus type 2b (PCV2b) in commercial pigs, with or without prior vaccination for PRRS. J Anim Sci.

[16] Harris N, Kunicka J, Kratz A (2005). The ADVIA 2120 hematology system: flow cytometry-based analysis of blood and body fluids in the routine hematology laboratory. Laboratory hematology: official publication of the International Society for Laboratory Hematology.

[17] Debey S, Schoenbeck U, Hellmich M, Gathof BS, Pillai R, Zander T, Schultze JL (2004). Comparison of different isolation techniques prior gene expression profiling of blood derived cells: impact on physiological responses, on overall expression and the role of different cell types. The pharmacogenomics journal.

[18] Raghavachari N, Xu X, Munson PJ, Gladwin MT (2009). Characterization of whole blood gene expression profiles as a sequel to globin mRNA reduction in patients with sickle cell disease. PLoS One.

[19] Putz AM, Harding JCS, Dyck MK, Fortin F, Plastow GS, Dekkers JCM (2018). Novel resilience phenotypes using feed intake data from a natural disease challenge model in wean-to-finish pigs. Front Genet.

[20] Andrews S. FASTQC. A quality control tool for high throughput sequence data. 2010.

[21] Dobin A, Davis CA, Schlesinger F, Drenkow J, Zaleski C, Jha S, Batut P, Chaisson M, Gingeras TR (2013). STAR: ultrafast universal RNA-seq aligner. Bioinformatics (Oxford, England).

[22] Anders S, Pyl PT, Huber W (2015). HTSeq--a Python framework to work with high-throughput sequencing data. Bioinformatics (Oxford, England).

