# Supplementary material for: The effects of a globin blocker on the resolution of 3’mRNA sequencing data in porcine blood
Source: BMC Genomics. 2019 Oct 15;20:741. doi: 10.1186/s12864-019-6122-2 (PMC6794815; doi:10.1186/s12864-019-6122-2)
Supplement: Supplementary file 1 — Additional file 1: Table S1. The effects of inclusion of a globin blocker in library construction on the composition of reads mapped to genes in data set 1. Table S2. The number of expressed genes in libraries created with and without globin blocker in data set 1. Table S3. The list of genes that were identified as expressed in the library constructed without a globin blocker but not in libraries constructed with the globin blocker at concentration C2 in sample A. Table S4. Summary statistics for QuantSeq 3′ mRNA sequencing data from biological replicates in data set 2. Table S5. Summary statistics for QuantSeq 3′ mRNA sequencing of libraries created with the globin blocker in data set 3 [file 12864_2019_6122_MOESM1_ESM.docx]

**Table S1.** The effects of inclusion of a globin blocker in library construction on the composition of reads mapped to genes in data set 1.

|  | Non-globin block | Globin block | *P*-value |
| --- | --- | --- | --- |
| Globin genes (%) | 57.96 (5.44) | 19.47 (3.99) | 0.005 |
| Hemoglobin alpha (%) | 22.54 (2.60) | 15.18 (1.91) | 0.085 |
| Hemoglobin beta (%) | 35.42 (3.38) | 4.27 (2.48) | 0.002 |
| Non-globin genes (%) | 10.86 (2.27) | 22.21 (1.66) | 0.016 |

The numbers in parentheses refer to standard errors.

**Table S2.** The number of expressed genes in libraries created with and without globin blocker in data set 1.

|  |  | Number of genes |  |  |  |
| --- | --- | --- | --- | --- | --- |
| Biological  replicate | Globin Blocker  concentration | Expressed ^a^ in both NGB and GB | Expressed only in GB | Expressed only in NGB | Not expressed in  either NGB or GB |
| A | C1 | 8,380 | 1,232 | 57 | 16,211 |
|  | C2 | 8,397 | 1,295 | 40 | 16,148 |
|  | C3 | 8,395 | 1,121 | 42 | 16,322 |
|  | C4 | 8,376 | 894 | 61 | 16,549 |
| B | C3 | 6,941 | 1,606 | 149 | 17,184 |

GB, globin blocker; NGB, non-GB.

^a^ Threshold of expressed gene: > 5 reads counts per 10 million of total clean read counts

**Table S3.** The list of genes that were identified as expressed in the library constructed without a globin blocker but not in libraries constructed with the globin blocker at concentration C2 in sample A.

|  | Counts per 10 million of total clean read counts | |
| --- | --- | --- |
| Gene ID | NGB | GB with concentration C2 |
| ENSSSCG00000025924 | 6.85 | 4.74 |
| ENSSSCG00000013582 | 5.96 | 4.74 |
| ENSSSCG00000005083 | 5.96 | 4.74 |
| ENSSSCG00000014003 | 5.36 | 4.74 |
| ENSSSCG00000010923 | 5.07 | 4.74 |
| ENSSSCG00000018682 | 5.66 | 4.42 |
| ENSSSCG00000012378 | 5.36 | 4.42 |
| ENSSSCG00000037986 | 8.64 | 4.11 |
| ENSSSCG00000007700 | 5.96 | 4.11 |
| ENSSSCG00000007366 | 5.66 | 4.11 |
| ENSSSCG00000012818 | 5.66 | 4.11 |
| ENSSSCG00000038412 | 5.36 | 4.11 |
| ENSSSCG00000039650 | 5.36 | 4.11 |
| ENSSSCG00000009879 | 5.07 | 4.11 |
| ENSSSCG00000010077 | 5.07 | 4.11 |
| ENSSSCG00000038487 | 5.07 | 4.11 |
| ENSSSCG00000009524 | 7.75 | 3.79 |
| ENSSSCG00000003753 | 5.36 | 3.79 |
| ENSSSCG00000021601 | 5.36 | 3.79 |
| ENSSSCG00000006862 | 5.07 | 3.79 |
| ENSSSCG00000002138 | 7.45 | 3.48 |
| ENSSSCG00000040682 | 5.96 | 3.48 |
| ENSSSCG00000017515 | 5.66 | 3.48 |
| ENSSSCG00000007909 | 5.36 | 3.48 |
| ENSSSCG00000008233 | 5.07 | 3.48 |
| ENSSSCG00000011404 | 6.56 | 3.16 |
| ENSSSCG00000038671 | 7.45 | 2.84 |
| ENSSSCG00000032170 | 5.66 | 2.84 |
| ENSSSCG00000007708 | 5.96 | 2.53 |
| ENSSSCG00000025408 | 5.96 | 2.53 |
| ENSSSCG00000001409 | 5.96 | 2.21 |
| ENSSSCG00000012780 | 5.66 | 2.21 |
| ENSSSCG00000004236 | 5.36 | 2.21 |
| ENSSSCG00000038474 | 5.07 | 2.21 |
| ENSSSCG00000004479 | 5.96 | 1.90 |
| ENSSSCG00000010600 | 5.07 | 1.90 |
| ENSSSCG00000036629 | 5.07 | 1.90 |
| ENSSSCG00000000359 | 6.26 | 1.58 |
| ENSSSCG00000035364 | 5.07 | 1.26 |
| ENSSSCG00000026317 | 5.96 | 0.32 |

**Table S4.** Summary statistics for QuantSeq 3′ mRNA sequencing data from biological replicates in data set 2.

|  | Non-globin block samples (n=184) | | | |  | Globin block samples (n=189) | | | |
| --- | --- | --- | --- | --- | --- | --- | --- | --- | --- |
| Sample | Mean | SD | Min | Max |  | Mean | SD | Min | Max |
| Total reads (millions) | 3.38 | 1.13 | 0.76 | 7.53 |  | 3.28 | 1.07 | 0.81 | 9.59 |
| Aligned reads (%) | 98.75 | 0.53 | 94.45 | 99.39 |  | 98.84 | 0.22 | 97.24 | 99.29 |
| Unique-mapping reads (%) | 87.81 | 3.59 | 74.23 | 92.57 |  | 84.18 | 3.85 | 60.64 | 89.70 |
| Gene reads (%) | 66.77 | 8.33 | 42.37 | 80.91 |  | 54.38 | 8.78 | 14.24 | 73.42 |
| Globin reads (%) | 45.25 | 10.69 | 13.82 | 63.85 |  | 19.30 | 7.60 | 2.60 | 37.9 |
| Hemoglobin alpha reads (%) | 10.84 | 2.30 | 3.13 | 15.7 |  | 12.43 | 4.27 | 1.99 | 23.37 |
| Hemoglobin beta reads (%) | 34.4 | 9.24 | 9.98 | 50.44 |  | 6.85 | 4.00 | 0.57 | 18.78 |
| Non-globin reads (%) | 21.53 | 3.62 | 13.47 | 39.72 |  | 35.10 | 5.10 | 11.68 | 60.56 |

**Table S5.** Summary statistics for QuantSeq 3′ mRNA sequencing of libraries created with the globin blocker in data set 3.

|  | Globin block samples (n=86) | | | |
| --- | --- | --- | --- | --- |
| Statistics | Mean | SD | Min | Max |
| Total reads (millions) | 7.29 | 1.34 | 4.07 | 10.45 |
| Aligned reads (%) | 98.67 | 0.40 | 95.86 | 99.34 |
| Unique-mapping reads (%) | 82.07 | 2.83 | 69.25 | 85.21 |
| Gene reads (%) | 43.89 | 6.70 | 27.03 | 62.36 |
| Globin reads (%) | 10.70 | 4.61 | 2.26 | 23.81 |
| Hemoglobin alpha reads (%) | 7.75 | 3.73 | 1.60 | 21.12 |
| Hemoglobin beta reads (%) | 2.95 | 1.72 | 0.35 | 7.39 |
| Non-globin reads (%) | 33.19 | 4.12 | 18.10 | 42.22 |
