# Supplementary material for: The effects of a globin blocker on the resolution of 3’mRNA sequencing data in porcine blood
Source: BMC Genomics. 2019 Oct 15;20:741. doi: 10.1186/s12864-019-6122-2 (PMC6794815; doi:10.1186/s12864-019-6122-2)
Supplement: Supplementary file 2 — Additional file 2: Figure S1. Scatter plots of scaled counts* between NGB and GB. (a) relationship between NGB and GB with C1 in sample A, (b) relationship between NGB and GB with C3 in sample A, (c) relationship between NGB and GB with C4 in sample A, (d) relationship between NGB and GB with C3 in sample B. *log2(count per 10 million + 1). Figure S2. Summary statistics for QuantSeq with or without GB at concentration C2. ALIGN, aligned reads; UNIQ, unique-mapping reads; GENE, reads mapped to all genes; HBA/HBB, reads mapped to HBA and HBB genes; Non-globin, reads mapped to non-HB genes. Figure S3. Distributions of HBA and HBB counts as a percentage of total reads in biological replicates by NGB and GB. Figure S4. Scatter plots of globin read percentage (%) with RIN and the number of clean reads in NGB (a and b) and GB (c and d) [file 12864_2019_6122_MOESM2_ESM.pptx]

## Slide 1
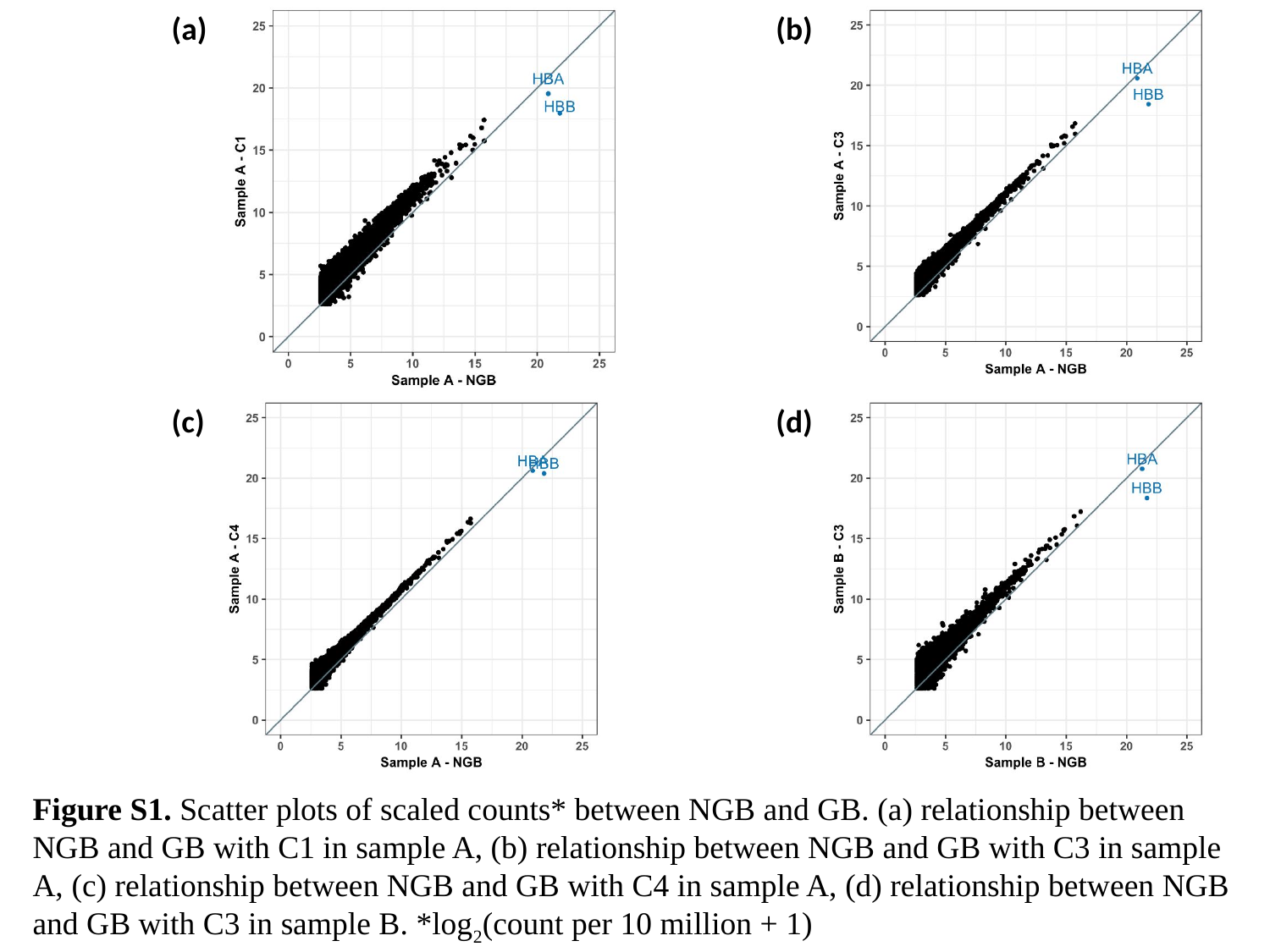

(a)
(b)
(c)
(d)
Figure S1. Scatter plots of scaled counts* between NGB and GB. (a) relationship between NGB and GB with C1 in sample A, (b) relationship between NGB and GB with C3 in sample A, (c) relationship between NGB and GB with C4 in sample A, (d) relationship between NGB and GB with C3 in sample B. *log2(count per 10 million + 1)

## Slide 2
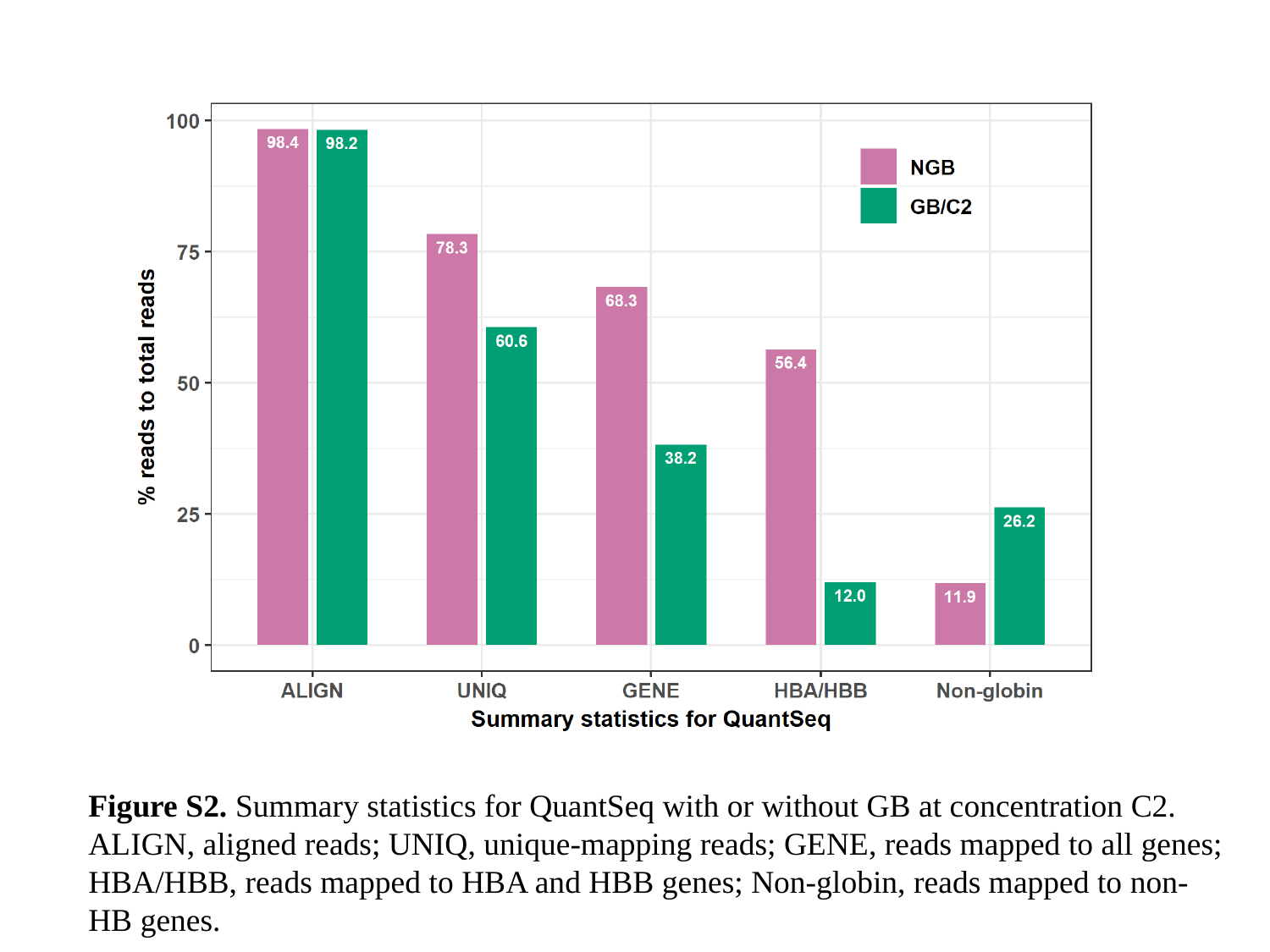

Figure S2. Summary statistics for QuantSeq with or without GB at concentration C2.
ALIGN, aligned reads; UNIQ, unique-mapping reads; GENE, reads mapped to all genes; HBA/HBB, reads mapped to HBA and HBB genes; Non-globin, reads mapped to non-HB genes.

## Slide 3
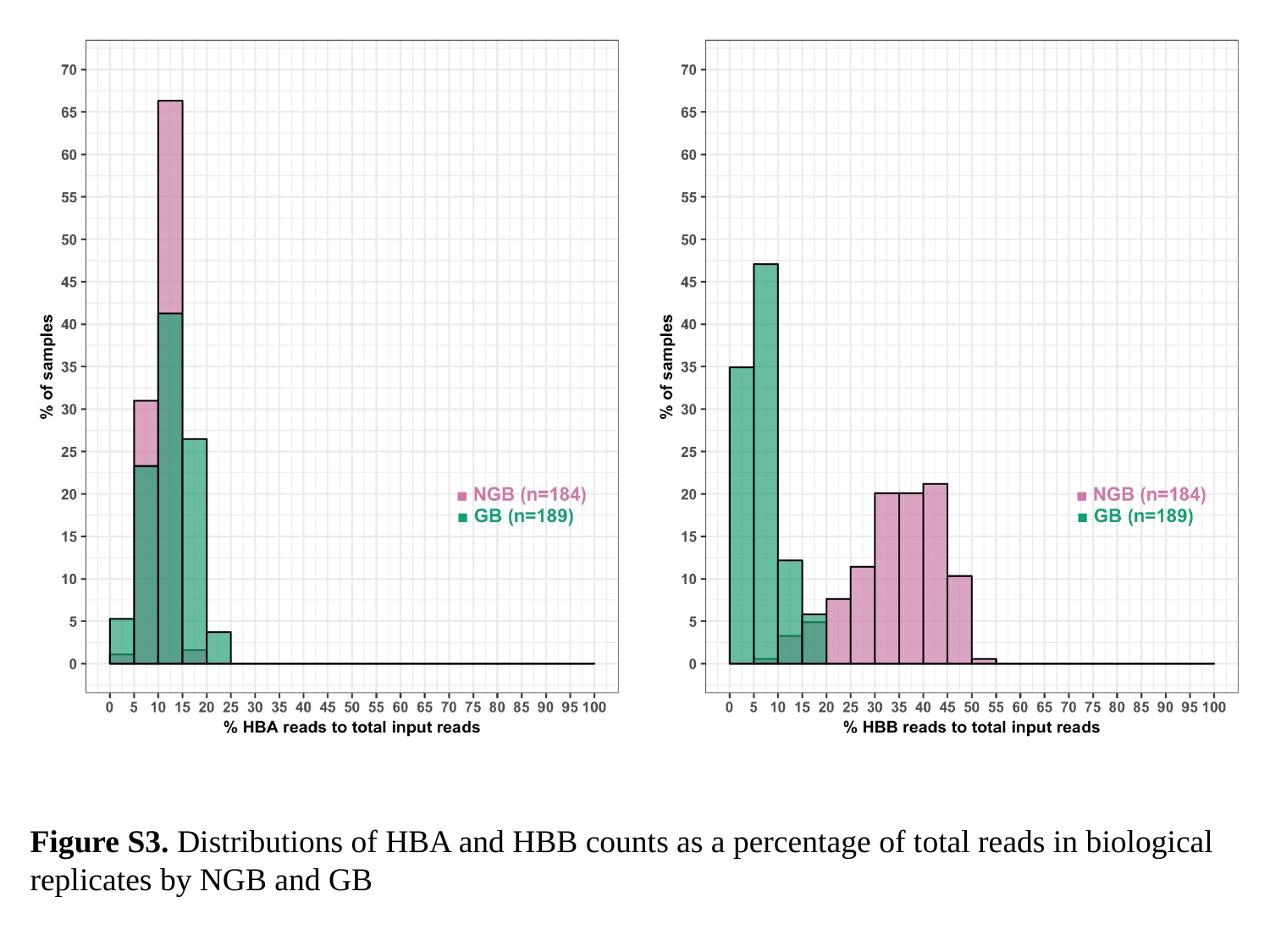

Figure S3. Distributions of HBA and HBB counts as a percentage of total reads in biological replicates by NGB and GB

## Slide 4
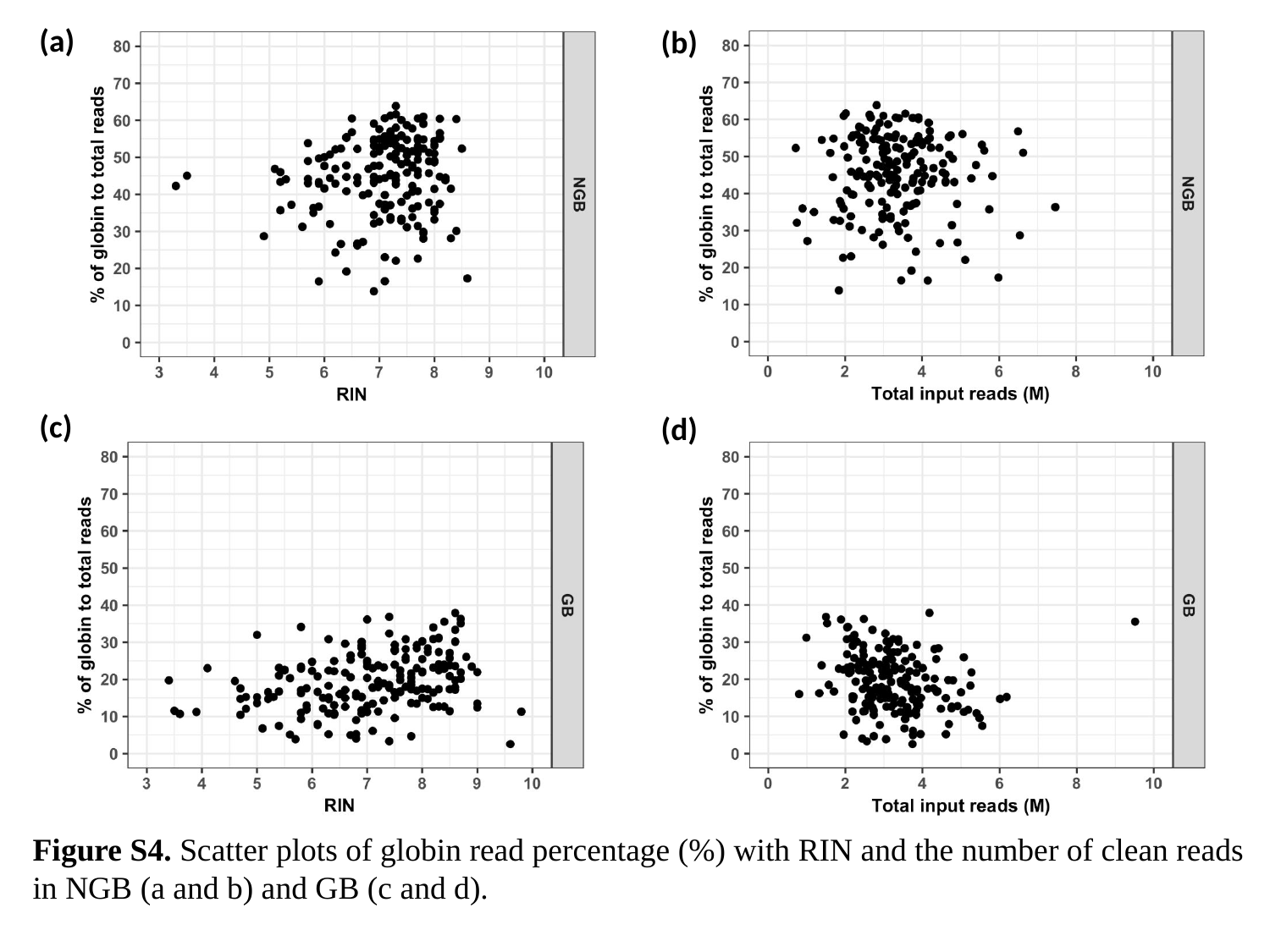

(a)
(b)
(c)
(d)
Figure S4. Scatter plots of globin read percentage (%) with RIN and the number of clean reads in NGB (a and b) and GB (c and d).
